# Rotational thrombelastometry (ROTEM) improves hemostasis assessment compared to conventional coagulation test in ACLF and Non-ACLF patients

**DOI:** 10.1186/s12876-020-01413-w

**Published:** 2020-08-17

**Authors:** Jessica Seeßle, Jan Löhr, Marietta Kirchner, Josefin Michaelis, Uta Merle

**Affiliations:** 1 Department of Gastroenterology, University Hospital Heidelberg, Im Neuenheimer Feld 410, 69120 Heidelberg, Germany; 2 Institute of Medical Biometry and Informatics, University Hospital Heidelberg, Heidelberg, Germany

**Keywords:** Acute-on-chronic liver failure (ACLF), Non-ACLF, Liver cirrhosis, Hemostasis, Rotational thrombelastometry (ROTEM), Conventional coagulation test (CCT)

## Abstract

**Background:**

Patients with liver cirrhosis typically exhibit abnormal coagulation parameters in conventional coagulation tests (CCTs). Rotational thromboelastometry (ROTEM) is a holistic blood coagulation assay. This method provides an insight into the global hemostatic capabilities and has been suggested to provide a better overview of the coagulation system in liver cirrhosis.

**Methods:**

The goal of this study was to examine hemostasis in patients with stable liver cirrhosis (Non-ACLF) and in acute-on-chronic liver failure (ACLF) by CCT and ROTEM including agreement of both tests and the prospective assessment of test performance based on clinical outcomes in ACLF patients. Therefore, ACLF patients were additionally subgrouped by bleeding events. Fifty-five Non-ACLF patients and twenty-two patients with ACLF were analysed in this prospective cohort study.

**Results:**

Coagulation parameters analysed by CCT were outside the normal range in Non-ACLF and ACLF patients, but were significantly more aberrant in ACLF patients. Non-ACLF patients analysed by ROTEM revealed parameters largely within the normal limits, while significantly more ROTEM parameters in ACLF patients were affected. Maximum clot firmness (MCF) was significantly divergent between both patient groups and correlated well with levels of fibrinogen and platelet count. Using Cohen’s Kappa coefficient κ, the strength of agreement between CCT and ROTEM analyses was determined to be fair for Non-ACLF patients and moderate for ACLF patients. Bleeding events occurred significantly more often in ACLF group with significantly reduced A10 and MCF.

**Conclusions:**

For assessing hemostasis in Non-ACLF and ACLF patients the underlying dataset shows advantages of ROTEM over CCT. A10 and MCF represent suitable prognostic parameters in predicting bleeding events in ACLF group.

## Background

Patients with liver cirrhosis have long been considered ‘auto-anticoagulated’ due to changes seen in conventional coagulation tests (CCTs). The commonly occurring laboratory findings corresponding to this alteration of hemostasis in cirrhotic patients are raised INR, prolonged aPTT and thrombocytopenia. However, these measurements are being questioned for their ability to correctly represent the in vivo hemostatic state of cirrhotic patients [1]. The main drawback of CCT is that it measures only the procoagulant activity but not the anticoagulant activity. In addition, samples for INR and aPTT measurements are being centrifuged and thus consist solely of a patient’s serum. This results in a lack of representation of the cellular parts of coagulation in these assays. The in vitro nature of CCT results in a lack of information about the vessel-bound parts of the coagulation system. In particular, the lack of information about thrombomodulin, which massively increases thrombin-induced activation of protein C and thus causes anticoagulation, leads to different results compared with in vivo conditions [2]. Therefore, the interaction between procoagulant (factors II, VII, IX, X) and anticoagulant hemostatic components (protein C and S) and platelets cannot be assessed with CCT. In hepatic coagulopathy, vitamin K-dependent factors II, VII, IX and X and vitamin K-dependent anticoagulant proteins C and S are decreased [3]. In addition, fibrinogen and factor V levels are decreased, while factor VIII and the von-Willebrand factor (v-Wf) are elevated by up to 200% of the reference value means [4]. In summary, in patients with liver cirrhosis, the loss of pro-hemostatic drivers is balanced by the loss of anti-hemostatic processes.

In contrast to CCT, a composite dynamic picture of the entire coagulation process is given by global viscoelastic tests. Thrombelastography, a whole blood coagulation assay developed by Hartert in 1948, provides an insight into the global hemostatic capabilities of a patient’s blood sample. Today there are two different systems readily available: thrombelastography (TEG) and rotational thrombelastometry (ROTEM). Both methods rely on Hartert’s principle of a pin and a cup containing the blood sample rotating relatively to each other to measure the strength of the blood clots. ROTEM allows to detect abnormalities in different components of the coagulation system by adding activator and inhibitor reagents to the citrated blood, in particular INTEM, EXTEM, FIBTEM, APTEM and NATEM.

Several studies have shown that liver cirrhosis patients with an impaired coagulation in CCT have a preserved or at least less impaired coagulation based on TEG results [1, 5–7]. Because of that, patients with cirrhosis are considered to be mostly in a delicately ‘balanced hemostasis’, although this balanced coagulation state is not depicted in CCT [8–10].

Acute-on-chronic liver failure (ACLF) is defined as acute deterioration of pre-existing, chronic liver disease, usually related to a precipitating event and associated with increased mortality at 3 months due to multi-system organ failure. ACLF is distinct from typical acute decompensation with respect to precipitating factors, age and the presence of acute systemic inflammation [1].

As patients with ACLF typically have a pronounced deterioration in coagulation according to CCT results, we questioned whether ROTEM measurements would also show preserved coagulation in this severely ill subgroup of liver cirrhosis patients. Therefore, we conducted a cross-sectional study to investigate the severity of coagulopathy in cirrhotic patients in a compensated stage that did not match the ACLF criteria (Non-ACLF) and in patients with ACLF. Coagulation was measured with CCT and ROTEM. We further examined the strength of agreement between CCT and ROTEM in Non-ACLF and ACLF patients and assessed prospectively the test performance based on clinical outcomes in ACLF patients.

## Methods

### Study population

In this prospective cohort study, we included all adult (age ≥ 18 years) liver cirrhosis patients who were admitted to the Department of Gastroenterology at the University Hospital of Heidelberg from 1st of July 2017 to 31st of December 2017 consecutively and who gave their written informed consent. Ethical approval was given by the Ethics Committee of University of Heidelberg. ACLF was defined as an acute deterioration of liver function in patients with chronic liver disease according to EASL/AASLD guidelines and classified by CLIF-consortium ACLF organ failure scores (CLIF-C OFs) as I°, II°, III° or Non-ACLF [11]. The score includes the following parameters: bilirubin, creatinine, encephalopathy, INR, blood pressure and oxygenation.. Adult patients aged 18 years or older with a diagnosis of cirrhosis were included. Cirrhosis was diagnosed by the presence of two or more of the following: I.) laboratory data; II.) radiological findings, including ultrasound, MRI or CT scan; III.) histological evidence by liver biopsy consistent with liver cirrhosis. Clinical and laboratory data including conventional coagulation tests (CCT) and ROTEM analysis, were collected within 24 h of admission. CCT performed included international normalized ratio (INR), prothrombin time (PT/Quick), activated partial thromboplastin time (aPTT), platelet count, fibrinogen, antithrombin III-activity, thrombin time, protein C, free protein S, factor V, factor VIII, von-Willebrand antigen and ADAMTS13, and were measured in the hospital central laboratory according to standard methods. ACLF patients were admitted because of acute-on-chronic liver failure according to ACLF criteria. Patient included in the Non-ACLF group were admitted for the following reasons: TACE (29.1%), check of TIPSS (18.2%), evaluation of liver transplantation (23.6%), transarterial chemoperfusion (5.5%), CT-guided biopsy of the liver (3.6%), gastrointestinal bleeding (3.6%), hepatic encephalopathy (3.6%), SIRT (1.8%), arterial bleeding of gluteal muscle (1.8%), TIPSS implantation (1.8%), ERCP (1.8%), ascites (1.8%), diagnostic angiography (1.8%) and tooth extraction (1.8%). Patients were followed for a median follow-up time of 21 months.

### Rotational thrombelastometry (ROTEM) assay

ROTEM was performed at 37 °C on a single instrument (ROTEM delta, TEM innovations). Briefly, 5 ml of citrated blood was subjected to ROTEM within 2 h of blood draw. A run time of 60 min was applied. For the present study, 5 tests (INTEM, EXTEM, FIBTEM, APTEM and NATEM) were carried out. Reagents provided by the manufacturer were used. For each of the tests, the blood was re-calcified with 20 nl 0.2 mol/l CaCl_2_ (star-TEM; Pentapharm, Munich, Germany) and activation of coagulation was performed with different agents:

#### INTEM

Contact pathway activation of coagulation with 20 nl of contact activator (partial thromboplastin–phospholipid from rabbit brain extract and ellagicacid, in-TEM; Pentapharm, Munich, Germany);

#### EXTEM

Tissue factor pathway activation of coagulation with 20 nl of tissue factor (TF, tissue thromboplastin from rabbit brain extract, ex-TEM; Pentapharm, Munich, Germany);

#### APTEM

TF plus 20 nl of aprotinin, plasmin-antagonist (ap-TEM; Pentapharm, Munich, Germany);

#### FIBTEM

TF plus inhibition of thrombocytes with 20 nl of cytochalasin (fib-TEM; Pentapharm, Munich, Germany).

For **NATEM**, the sample was also recalcified, but no activators of coagulation were added.

The analysed thrombelastometric parameters for NATEM, INTEM, EXTEM and APTEM were clotting time (CT): time in seconds from start of the analysis until detectable clotting; clot formation time (CFT): time in seconds from initiating clotting until an amplitude of graphical trace of 20 mm is established; maximal clot firmness (MCF): maximal amplitude (millimetres) of graphical trace of clot firmness; A10: clot firmness (millimetres) at the amplitude time point of 10 min after CT; α-angle: angle between the baseline and a tangent to the clotting curve through the 2 mm (CT/R) point. Maximum lysis (ML): reduction of clot firmness after MCF in relation to MCF. For FIBTEM, only MCF and A10 were investigated.

### Statistical analysis

Variables were described by median (interquartile range IQR) or frequencies. Statistical differences in the distribution of metric variables between two groups (Non-ACLF vs. ACLF, Bleeding vs. no bleeding and infection vs. no infection) were evaluated using the nonparametric Mann-Whitney U test due to small sample sizes in some of the groups and partly skewed distribution of variables. Statistical differences in proportions were assessed by chi-square test or Fisher’s exact test in case of small expectancy counts. The Spearman correlation coefficient was used for assessing correlations of two metric variables (*r* = 0.6–1.0 was considered as strong, *r* = 0.4–0.59 as moderate and *r* = 0.0–0.39 was considered a weak correlation). To measure agreement between CCT and ROTEM analyses (outside/inside the normal range), Cohen’s kappa coefficient was calculated. The strength of agreement was considered poor < 0.00, slight 0.00–0.20, fair 0.21–0.40, moderate 0.41–0.60, substantial 0.61–0.80 or almost perfect 0.81–1.0. Kaplan-Meier survival curves were plotted for Non-ACLF vs. ACLF groups and compared by means of the log-rank test. If possible, median survival time with 95% confidence interval was reported. Cox’s proportional hazard model was used to identify risk factors for overall survival. First, univariate models were applied and second, significant variables were included in a multivariable analysis separately for the ROTEM and CCT variables. *P*-values were interpreted descriptively, and a *p*-value of < 0.05 was considered statistically significant. All analyses were performed with IBM SPSS Statistics for Windows, Version 24.0, Armonk, NY: IBM Corp.)

## Results

### Assessment of CCT and ROTEM

#### Patient characteristics

Patient characteristics are outlined in Table 1. Of 77 patients with liver cirrhosis, 22 patients fulfilled the diagnostic criteria for ACLF at study inclusion. Age and gender were equally distributed between Non-ACLF and ACLF liver cirrhosis patients. In the Non-ACLF group, significantly more patients had hepatocellular carcinoma. This was due to the fact that admission for the transarterial chemoembolization procedure was often the reason for elective hospital admission in the Non-ACLF group. More patients in the ACLF group had an alcoholic cirrhosis etiology compared to the non-ACLF group (72.7% vs. 50.9%). In the ACLF group, significantly more patients had ascites grade ≥ 2 (86.4% vs. 34.5%, *p* < 0.0001), and hepatic encephalopathy > 2 (40.9% vs. 3.6%, *p* < 0.0001). Model for end-stage liver disease (MELD) score (25.5 vs. 11.0, *p* < 0.0001) and Child-Pugh score (12.0 vs. 2.0, *p* < 0.0001) were significantly higher in the ACLF group. In the ACLF group, most of the patients had Child-Pugh class C (81.8% vs. 1.8% in Non-ACLF group). In the ACLF group, significantly more patients were admitted to ICU (86.4% vs. 12.7%, *p* < 0.0001). In the ACLF group, 78.9% survived their ICU stay and 68.2% survived the hospital stay. In contrast, all patients with Non-ACLF survived the ICU and hospital stay.

**Table 1:** Demographics of study population. Median (IQR) or frequencies n (%) are shown with *p*-values for the group differences based on Mann-Whitney U test (a), Chi-square test (b) or Fisher’s exact test (c)

	Non-ACLF *n* = 55	ACLF *n* = 22	***p***-value
Age	59.0 (14.0)	56.0 (13.0)	0.42^a^
Male	37 (67.3%)	13 (59.1%)	0.50^b^
Etiology			
HCV	6 (10.9%)	1 (4.5%)	0.72^c^
HBV	5 (9.1%)	2 (9.1%)	
Alcohol	28 (50.9%)	16 (72.7%)	
Autoimmune	5 (9.1%)	1 (4.5%)	
NASH	3 (5.5%)	0 (0.0%)	
others	8 (14.5%)	2 (9.1%)	
Ascites grade ≥ 2	19 (34.5%)	19 (86.4%)	**< 0.0001**^**b**^
HE grade > 2	2 (3.6%)	9 (40.9%)	**< 0.0001**^**c**^
MELD score	11.0 (6.0)	25.5 (12.0)	**< 0.0001**^**a**^
MELD-Na^+^ score	12.0 (17.0)	26.0 (10.3)	**< 0.0001**^**a**^
Child-Pugh score	2.0 (1.0)	12.0 (3.25)	**< 0.0001**^**a**^
Child-Pugh class			
A	23 (41.8%)	1 (4.5%)	**< 0.0001**^**c**^
B	20 (36.4%)	3 (13.6%)	
C	12 (1.8%)	18 (81.8%)	
ACLF grade at admission			
0	55 (100%)	0 (0.0%)	**< 0.0001**^**c**^
1	0 (0.0%)	8 (36.4%)	
2	0 (0.0%)	10 (45.5%)	
3	0 (0.0%)	4 (18.2%)	
Bleeding events	3 (5.5%)	9 (40.9%)	**< 0.0001**^**c**^
Variceal bleeding	0 (0.0%)	5 (55.6%)	0.21^c^
Upper GI-bleeding	2 (3.6%)	1 (11.1%)	
Lower GI-bleeding	1 (1.8%)	1 (11.1%)	
Hematuria	0 (0.0%)	1 (11.1%)	
Spontaneous abdominal bleeding	0 (0.0%)	1 (11.1%)	
Infectious disease	9 (16.4%)	14 (63.6%)	**< 0.0001**^**c**^
Pneumonia	3 (5.5%)	6 (27.3%)	0.82^c^
Urinary tract infection	1 (1.8%)	2 (9.1%)	
Bacterial peritonitis	1 (1.8%)	3 (13.6%)	
Others/unknown	5 (9.1%)	4 (18.2%)	
Sepsis	0 (9.1%)	5 (22.7%)	**< 0.001**^**c**^
Hepatocellular carcinoma	25 (45.5%)	1 (4.5%)	**< 0.0001**^**c**^
Admission and Mortality			
ICU admission	7 (12.7%)	19 (86.4%)	**< 0.0001**^**c**^
Length of ICU admission (d)	0.0 (0.0)	3.5 (9.3)	**< 0.0001**^**a**^
Length of hospital admission (d)	6.0 (7.0)	17.0 (29.8)	**< 0.0001**^**a**^
Survived ICU	7 (100%)	12 (63.2%)	**< 0.0001**^**c**^
Survived hospital stay	55 (100%)	15 (68.2%)	**< 0.0001**^**b**^

*ACLF* acute-on-chronic liver failure, *HBV* hepatitis B, *HCV* hepatitis C, *d* days, *GI* gastrointestinal, *MELD* model of end stage liver disease, *HE* hepatic encephalopathy, *NASH* non-alcoholic fatty liver disease, *ICU* intensive care unit, *IQR* interquartile range

Bleeding occurred significantly more often in ACLF patients than in Non-ACLF patients (40.9% vs. 5.5%, *p* < 0.0001). None of the bleeding patients received any anticoagulant medication. In the ACLF group, 9 of 22 patients had a bleeding complication. The site of bleeding in ACLF patients was variceal bleeding in 5 patients, while gastric ulcer-related upper GI-bleeding, lower GI-bleeding, spontaneous intra-abdominal bleeding and hematuria occurred in 1 patient each.

Infectious diseases differed significantly between the groups (63.3% vs. 16.4% in Non-ACLF group, *p* < 0.001). Pneumonia (27.3% vs. 5.5%) and bacterial peritonitis (13.6% vs. 1.8%) were the most frequent causes. Five patients in the ACLF group developed a sepsis and no patient in the Non-ACLF group (*p* < 0.001).

#### Laboratory findings and Conventional Coagulation Tests (CCT) of study population

CCT showed major differences between the Non-ACLF and ACLF group (Table 2). PT (Quick) (50.0% vs. 70.0%, *p* = < 0.0001) and platelets (57/nl vs. 112/nl, *p* = 0.002) were significantly lower and INR (1.4 vs. 1.2, *p* = < 0.0001) and aPTT (31.8 s vs. 26.6 s, *p* = < 0.0001) were significantly higher in the ACLF group. Mean fibrinogen in both groups was within normal limits but was significantly lower in ACLF patients (1.6 g/l vs. 2.7 g/l, *p* = 0.001). Protein C (36.4% vs. 51.5%, *p* = 0.004), free protein S (52.9% vs. 69.4%, *p* = 0.007) and factor V (41.7% vs. 69.2%, *p* < 0.0001) were significantly lower in the ACLF group, whereas no difference was seen in factor VIII between the groups. Von-Willebrand antigen was clearly increased in both groups, but significantly higher in the ACLF group (411.0% vs. 294.8%, *p* = 0.003).

**Table 2:** Laboratory findings and blood coagulation tests of study population. Median (IQR) is shown with *p*-values for the group differences based on Mann-Whitney U test

	Limits of normal	Non-ACLF *n* = 55	% outside the limits of normal	ACLF *n* = 22	% outside the limits of normal	***p***-value
**Laboratory findings**						
Hemoglobin	13–17 g/dl	12.0 (4.1)	65.5%	8.6 (2.1)	95.5%	**< 0.0001**
Creatinine	0.6–1.2 mg/dl	0.9 (0.5)	18.2%	2.18 (1.6)	77.3%	**< 0.0001**
Bilirubin	< 1.0 mg/dl	1.3 (1.5)	63.6%	3.9 (8.9)	81.8%	**< 0.0001**
AST	< 46 U/l	46.5 (35.3)	47.3%	58.0 (53.0)	45.5%	0.78
ALT	< 50 U/l	30.5 (34.3)	24.1%	26.0 (31.0)	22.2%	0.60
GGT	< 60 U/l	94.0 (137.0)	67.3%	81.0 (103.5)	63.6%	0.30
Alkaline phosphatase	40–130 U/l	138.5 (110.3)	48.1%	106.0 (97.0)	27.8%	0.06
Albumin	30–50 g/l	36.2 (8.4)	17.3%	31.0 (5.7)	44.4%	**< 0.0001**
**Coagulation tests**						
INR	< 1.2	1.2 (0.2)	32.7%	1.4 (0.8)	81.8%	**< 0.0001**
PT (Quick)	70–120%	70.0 (20.0)	60.0%	50.0 (30.0)	54.5%	**< 0.0001**
aPTT	< 36 s	26.6 (4.7)	1.9%	31.8 (16.1)	31.8%	**< 0.0001**
Platelet count	150–440/nl	112.0 (85.0)	7.3%	57.0 (71.3)	31.8%	**0.002**
Fibrinogen	1.8–3.5 g/l	2.7 (1.4)	14.5%	1.6 (1.2)	59.1%	**0.001**
Antithrombin III-activity	80–120%	71.0 (32.0)	67.3%	42.1 (33.0)	95.5%	**< 0.0001**
Thrombin time	< 22 s	18.6 (1.9)	1.8%	20.5 (5.45)	27.3%	0.02
Protein C	60–120%	51.5 (36.0)	67.3%	36.4 (31.0)	77.3%	**0.004**
Protein S (free)	> 80%	69.4 (24.0)	65.5%	52.9 (33.0)	81.8%	**0.007**
Factor V	70–120%	69.2 (44.0)	49.1%*	41.7 (30.0)	90.9%	**< 0.0001**
Factor VIII	80–120%	242.2 (81.1)	100%	249.3 (128.0)	95.5%	0.98
von-Willebrand antigen	70–120%	294.8 (262.0)	100%	411.0 (259.0)	100%	**0.003**
ADAMTS13 activity	40–130%	100.0 (15.0)	2.0%	81.0 (38.0)	9.5%	**0.004**

*ACLF* acute-on-chronic liver failure, *ADAMTS13* desintegrin and metalloprotease with thrombospondin-1-like domains 13, *ALT* alanine aminotransferase, *AST* aspartate aminotransferase, *GGT* gamma GT, *INR* international normalized ratio, *IQR* interquartile range, *aPTT* activated partial thromboplastin time, *PT* prothrombin time

#### ROTEM assay of study population

ROTEM testing was performed with NATEM, INTEM, EXTEM, FIBTEM and APTEM (Table 3). Major differences were observed between the groups. In INTEM, EXTEM and APTEM, significant differences were shown between CFT, MCF, A10 and α-angle in ACLF and Non-ACLF patients. In FIBTEM, means of MCF, A10 and α-angle in both groups were in the normal range but were significantly lower for all three parameters in the ACLF group. Interestingly, in NATEM analysis, none of the parameters differed significantly between the groups. In summary, the Non-ACLF group showed a significantly shorter CFT, CT and higher MCF compared to ACLF patients.

**Table 3:** ROTEM analysis of study population. Median (IQR) is shown with *p*-values for the group differences based on Mann-Whitney U test

	Limits of normal^**a**^	Non-ACLF *n* = 55	% outside the limits of normal	ACLF *n* = 22	% outside the limits of normal	***p***-value
**NATEM**						
CT	254–837 s	549.0 (185.0)	3.7%	650.0 (237.5)	19%	**0.01**
CFT	72–357 s	182.0 (124.0)	5.6%	224.0 (114.4)	9.5%	0.42
MCF	46–69 mm	49.0 (16.0)	25.9%	43.0 (16.5)	38.1%	0.17
A10	–	40.0 (16.5)	–	34.0 (14.5)	–	0.23
α-angle	39–75°	57.0 (14.5)	5.7%	53.0 (10.5)	0%	0.40
**INTEM**						
CT	100–240 s	185.0 (32.0)	9.1%	209.0 (57.0)	19%	**0.01**
CFT	30–110 s	112.0 (76.0)	47.3	166.0 (112.5)	76.2%	**0.04**
MCF	50–72 mm	52.0 (16.0)	7.3%	44.0 (16.5)	33.3%	**0.048**
A10	44–66 s	45.0 (14.0)	43.6%	36.0 (13.5)	76.2%	**0.03**
α-angle	70–83°	71.0 (8.0)	41.8%	65.0 (12.0)	61.9%	**0.04**
**EXTEM**						
CT	38–79 s	61.0 (11.0)	3.6%	72.0 (17.25)	22.7%	**< 0.0001**
CFT	34–159 s	103.0 (63.0)	23.6%	173.5 (126.5)	63.6%	**0.008**
MCF	50–72 mm	54.0 (15.0)	5.5%	42.5 (14.3)	27.3%	**0.005**
A10	43–65 s	47.0 (17.0)	29.1%	35.5 (14.0)	77.3%	**0.004**
α-angle	63–83°	74.0 (10.0)	10.9%	65.0 (15.5)	40.1%	**0.001**
**FIBTEM**						
MCF	9–25 mm	16.0 (8.5)	5.5%	13.0 (9.3)	31.8%	**< 0.0001**
A10	7–23 s	15.0 (7.0)	1.8%	11.5 (9.8)	13.6%	**< 0.0001**
α-angle	–	75.0 (9.0)	–	70.5 (14.5)	–	**0.07**
**APTEM**						
CT	35–80 s	62.0 (10.0)	7.3%	64.5 (18.0)	18.2%	0.08
CFT	35–160 s	99.0 (106.0)	29.1%	181.5 (98.3)	59.1%	**0.02**
MCF	53–72 mm	55.0 (15.0)	3.6%	43.5 (15.8)	27.3%	**0.005**
A10	–	48.0 (16.0)	–	35.0 (13.3)	–	**0.007**
α-angle	–	74.0 (12.0)	–	63.5 (19.5)	–	**0.002**

*A10* amplitudes at 10 min, *ACLF* acute-on-chronic liver failure, *CFT* clot formation time, *CT* clotting time, *IQR* interquartile range, *MCF* maximum clot firmness, *ML* maximal lysis

^a^by the manufacturer

#### Comparison of CCT and corresponding variables of ROTEM analysis

When comparing ROTEM with CCT, ROTEM measurements depicted a normal coagulation state more frequently than CCT in Non-ACLF and ACLF patients. In addition, based on ROTEM results, ACLF patients showed a normal, ‘rebalanced’ coagulation state less frequently than Non-ACLF patients (Table 4 A-D).

**Table 4:** Comparison of corresponding variables of CCT and ROTEM analysis. Agreement is indicated by Cohen’s kappa κ with corresponding *p*-value

**A**					
			**Non-ACLF** *n* = 55	**ACLF** *n* = 22	
**CT EXTEM**		<80s	96.4%	81.8%	
		> 80s	3.6%	18.2%	
**PT (Quick)**		> 50%	80%	45.5%	
		< 50%	20%	54.6%	
		**Non-ACLF** *n* = 55		**ACLF** *n* = 22	
**CT EXTEM**		<80s	>80s	<80s	>80s
**PT (Quick)**	> 50%	97.7% (43/44)	2.3% (1/44)	100% (10/10)	0.0% (0/10)
	< 50%	90.9% (10/11)	9.1% (1/11)	66.7% (1/12)	33.3% (4/12)
	κ **/** p	0.098 / 0.28		0.313 / 0.04	
**B**					
			**Non-ACLF** *n* = 55	**ACLF** *n* = 22	
**CT INTEM**		< 240 s	90.7%	81%	
		> 240 s	9.3%	19%	
**aPTT**		< 36 s	98.1%	66.7%	
		> 36 s	1.9%	33.3%	
		**Non-ACLF** *n* = 55		**ACLF** *n* = 22	
**CT INTEM**		< 240 s	> 240 s	< 240 s	> 240 s
**aPTT**	< 36 s	92.5% (50/54)	7.5% (4/54)	100% (14/14)	0.0% (0/14)
	> 36 s	0.0% (0/1)	100% (1/1)	42.9% (3/7)	57.1% (4/7)
	κ **/** p	0.31 / 0.02		0.64 / 0.002	
**C**					
			**Non-ACLF** *n* = 55	**ACLF** *n* = 22	
**MCF EXTEM**		> 40 mm	94.5%	81.8%	
		< 40 mm	5.5%	18.2%	
**Platelets**		> 50/nl	92.7%	68.2%	
		< 50/nl	7.3%	31.8%	
		**Non-ACLF** *n* = 55		**ACLF** *n* = 22	
**MCF EXTEM**		> 40 mm	< 40 mm	> 40 mm	< 40 mm
**Platelets**	> 50/nl	96.1% (50/52)	3.9% (2/52)	86.7% (13/15)	13.3% (2/15)
	< 50/nl	75% (3/4)	25% (1/4)	42.9% (3/7)	57.1% (4/7)
	κ **/** p	0.24 / 0.07		0.46 / 0.03	
**D**					
		**Non-ACLF** *n* = 55		**ACLF** *n* = 22	
**MCF FIBTEM**	> 9 mm	94.5%		68.2%	
	< 9 mm	5.5%		31.8%	
**Fibrinogen**	> 1.6 g	89.1%		50%	
	< 1.6 g	10.9%		50%	
		**Non-ACLF** *n* = 55		**ACLF** *n* = 22	
**MCF FIBTEM**		> 9 mm	< 9 mm	> 9 mm	< 9 mm
**Fibrinogen**	> 1.6 g	95.9% (48/50)	4.1% (2/50)	90.9% (10/11)	9.1% (1/11)
	< 1.6 g	83.3% (5/6)	16.7% (1/6)	45.5% (5/11)	54.5% (6/11)
	κ **/** p	0.16 / 0.20		0.46 / 0.02	

*ACLF* acute-on-chronic liver failure, *aPTT* activated partial thromboplastin time, *CT* clotting time; MCF. maximum clot firmness

##### CT EXTEM versus PT (quick)

In 18.2% of patients with ACLF and in 3.6% of Non-ACLF patients, CT EXTEM was pathological (> 80s), whereas PT (Quick) was pathological (< 50%) in 54.6% of patients with ACLF and 20% of Non-ACLF patients. A pathological PT (Quick) but a normal CT EXTEM was found in 66.7% of patients with ACLF and 90.9% of non-ACLF patients (Table 4A).

##### CT INTEM versus aPTT

In 19.0% of ACLF patients and in 9.3% of non-ACLF patients, CT INTEM was increased (> 240 s), while aPTT (> 36 s) was prolonged in 33.3% of ACLF patients and in only 1.9% in the Non-ACLF group. Prolonged aPTT and normal CT INTEM was seen in 42.9% of patients with ACLF and none of Non-ACLF patients (Table 4B).

##### MCF EXTEM versus platelets

In 18.2% of ACLF patients and in 5.5% of non-ACLF patients, MCF EXTEM was < 40 mm. Platelet count was < 50/nl in 31.8% of ACLF patients and in 7.3% of Non-ACLF patients. In all, 42.9% of patients with ACLF and 75% of Non-ACLF patients had platelets < 50/nl and normal MCF EXTEM (Table 4C).

##### MCF FIBTEM versus fibrinogen

Reduced MCF FIBTEM (< 9 mm) was found in 31.8% of ACLF patients and 5.5% of Non-ACLF patients. Fibrinogen was below 1.6 g/l in 50% of ACLF patients and in 10.9% of Non-ACLF patients. 45.5% of patients with ACLF and 83.3% of the Non-ACLF patients had low fibrinogen levels and normal MCF FIBTEM (Table 4D).

To assess the strength of agreement between CCT and ROTEM parameters, the kappa coefficient was calculated (Table 4 A-D). In the ACLF group, the lowest agreement between CCT and ROTEM results was seen for CT EXTEM and PT, and moderate agreement was seen for MCF EXTEM/platelets (k/p: 0.46/0.03) and MCF FIBTEM/fibrinogen (k/p: 0.46/0.02).

##### Spearman correlation for CTT and ROTEM parameters

The Spearman correlation coefficient was analysed for CCT and ROTEM parameters in Non-ACLF and ACLF patients (Table 5 A-D). MCF (NATEM, INTEM, EXTEM and FIBTEM) showed the strongest correlation with fibrinogen and platelets in both groups. The correlation of CCT with CT and CFT (NATEM, INTEM, EXTEM and FIBTEM) was weaker.

**Table 5:** Spearman correlation coefficient r with corresponding *p*-value in Non-ACLF and ACLF patients

	Non-ACLF *n* = 55 r / p			ACLF *n* = 22 r / p		
**A**						
**NATEM**	**CT**	**CFT**	**MCF**	**CT**	**CFT**	**MCF**
**PT (Quick)**	**0.29 / 0.04**	− 0.04 / 0.76	0.25 / 0.07	− 0.05 / 0.71	0.32 / 0.83	− 0.05 / 0.16
**INR**	**−0.28 / 0.04**	0.06 / 0.68	−0.26 / 0.05	− 0.06 / 0.81	0.08 / 0.75	− 0.32 / 0.16
**aPTT**	−0.07 / 0.64	**0.28 / 0.04**	**− 0.34 / 0.01**	0.14 / 0.55	0.27 / 0.24	− 0.40 / 0.08
**Platelets**	−0.01 / 0.93	**−0.55 / < 0.0001**	**0.71 / < 0.0001**	−0.36 / 0.11	**− 0.47 / 0.03**	**0.62 / 0.003**
**Fibrinogen**	0.01 / 0.92	**−0.36 / 0.008**	**0.53 / < 0.0001**	− 0.02 / 0.92	− 0.41 / 0.06	**0.68 / 0.001**
**Antithrombin III-activity**	**0.31 / 0.02**	−0.20 / 0.15	**0.45 / 0.001**	−0.04 / 0.87	− 0.31 / 0.17	**0.65 / 0.001**
**Factor V**	0.14 / 0.33	**−0.30 / 0.03**	**0.45 / 0.001**	0.17 / 0.45	−0.11 / 0.64	**0.43 / 0.05**
**Factor VIII**	−0.25 / 0.07	**−0.42 / 0.002**	**0.29 / 0.04**	−0.37 / 0.10	**− 0.52 / 0.02**	**0.50 / 0.02**
**B**						
**INTEM**	**CT**	**CFT**	**MCF**	**CT**	**CFT**	**MCF**
**PT (Quick)**	−0.08 / 0.56	**− 0.31 / 0.02**	**0.44 / 0.001**	**−0.54 / 0.012**	− 0.13 / 0.58	0.21 / 0.36
**INR**	0.09 / 0.52	**0.33 / 0.01**	**−0.46 / < 0.0001**	**0.56 / 0.009**	0.12 / 0.60	−0.22 / 0.34
**aPTT**	**0.44 / 0.001**	**0.32 / 0.02**	**−0.42 / 0.002**	**0.637 / 0.002**	0.21 / 0.37	−0.26 / 0.26
**Platelet count**	−0.22 / 0.11	**− 0.79 / < 0.0001**	**0.78 / < 0.0001**	− 0.05 / 0.82	**−0.80 / < 0.0001**	**0.73 / < 0.0001**
**Fibrinogen**	**−0.32 / 0.02**	**− 0.61 / < 0.0001**	**0.68 / < 0.0001**	**−0.44 / 0.047**	**− 0.45 / 0.04**	**0.60 / 0.004**
**Antithrombin III-activity**	**−0.27 / 0.045**	**− 0.515 / < 0.0001**	**0.63 / < 0.0001**	**− 0.52 / 0.02**	**− 0.44 / 0.047**	**0.53 / 0.01**
**Factor V**	− 0.26 / 0.05	**− 0.48 / < 0.0001**	**0.56 / < 0.0001**	− 0.37 / 0.10	− 0.34 / 0.14	0.36 / 0.11
**Factor VIII**	− 0.26 / 0.06	**− 0.34 / 0.01**	**0.27 / 0.04**	− 0.07 / 0.76	**− 0.50 / 0.02**	0.43 / 0.05
**C**						
**EXTEM**	**CT**	**CFT**	**MCF**	**CT**	**CFT**	**MCF**
**PT (Quick)**	−0.01 / 0.94	**− 0.29 / 0.03**	**0.44 / 0.001**	**−0.4 / 0.02**	− 0.20 / 0.38	0.29 / 0.19
**INR**	0.003 / 0.98	**0.31 / 0.02**	**−0.46 / < 0.0001**	**0.49 / 0.02**	0.23 / 0.31	−0.32 / 0.15
**aPTT**	0.03 / 0.83	0.23 / 0.10	**−0.40 / 0.003**	**0.53 / 0.01**	0.27 / 0.23	−0.35 / 0.11
**Platelet count**	0.10 / 0.47	**−0.75 / < 0.0001**	**0.76 / < 0.0001**	**− 0.58 / 0.005**	**− 0.74 / < 0.0001**	**0.73 / < 0.0001**
**Fibrinogen**	0.08 / 0.54	**− 0.613 / < 0.0001**	**0.70 / < 0.0001**	**− 0.46 / 0.03**	**− 0.59 / 0.004**	**0.67 / 0.001**
**Antithrombin III-activity**	**0.28 / 0.04**	**− 0.50 / < 0.0001**	**0.64 / < 0.0001**	**−0.67 / 0.001**	**− 0.46 / 0.03**	**0.57 / 0.006**
**Factor V**	0.07 / 0.64	**−0.42 / 0.001**	**0.56 / < 0.0001**	**−0.47 / 0.03**	− 0.32 / 0.15	0.40 / 0.06
**Factor VIII**	−0.10 / 0.48	**−0.31 / 0.02**	0.25 / 0.07	−0.21 / 0.35	− 0.46 / 0.03	0.42 / 0.05
**D**						
**FIBTEM**	**MCF**			**MCF**		
**PT (Quick)**	**0.36 / 0.007**			**0.48 / 0.02**		
**INR**	**−0.38 / 0.005**			**−0.48 / 0.02**		
**aPTT**	**−0.36 / 0.008**			**−0.52 / 0.01**		
**Platelet count**	**0.47 / < 0.0001**			**0.52 / 0.013**		
**Fibrinogen**	**0.73 / < 0.0001**			**0.66 / 0.001**		
**Antithrombin III-activity**	**0.58 / < 0.0001**			**0.65 / 0.001**		
**Factor V**	**0.53 / < 0.0001**			**0.69 / < 0.0001**		
**Factor VIII**	**0.36 / 0.007**			0.34 / 0.12		

*ACLF* acute-on-chronic liver failure, *INR* international normalized ratio, *aPTT* activated partial thromboplastin time

### Assessment of outcomes

#### Survival

**Fig. 1:**
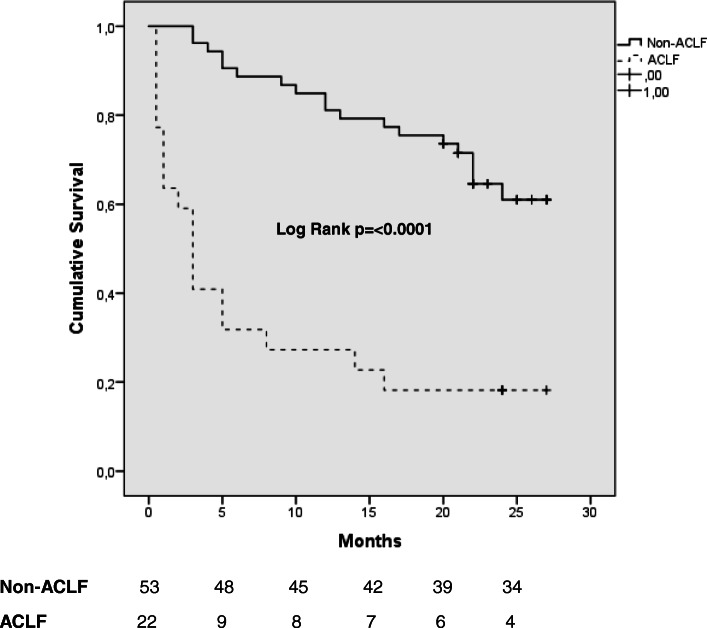
Kaplan-Meier’s analysis of survival between the study groups

All Non-ACLF patients (100%) and fifteen ACLF patients (68.2%) survived hospital stay (*p* = < 0.0001). Kaplan-Meier’s curve analysis showed significant survival differences between the groups (*p* = < 0.0001, Fig. 1). Within follow up time of 21 months nineteen patients (35.8%) in Non-ACLF group and eighteen patients in ACLF group (81.8%) died. Median survival in ACLF group was 3 months (95% CI: 1.9–4.1).

Table 6 shows the results of Cox regression analysis in relation to survival. PT, aPTT, INR, factor V, EXTEM CFT and EXTEM alpha angle were associated with survival (Table 6A). Multivariable analysis Cox regression analysis is outlined in Table 6 B + C. No significant differences were observed.

**Table 6:** Uni- (A) and multivariable analysis (B + C) according to Cox’s proportional hazard model

Variables	HR	(95% CI)	***p***-value
**A**			
**CCT**			
PT (Quick)	0.018	0.003–0.119	**< 0.0001**
INR	5.041	2.429–10.462	**< 0.0001**
aPTT	1.039	1.018–1.060	**< 0.0001**
Platelets	0.998	0.994–1.002	0.30
Fibrinogen	0.787	0.579–1.068	0.12
Factor V	0.219	0.064–0.754	**0.02**
**ROTEM**			
EXTEM CFT	1.004	1.001–1.007	**0.009**
EXTEM MCF	0.969	0.939–1.000	0.050
EXTEM alpha	0.957	0.926–0.988	**0.008**
**B**			
**CCT**			
INR	2.262	0.562–9.112	0.25
aPTT	1.00	0.965–1.036	0.99
Factor V	0.95	0.219–4.163	0.95
**C**			
**ROTEM**			
EXTEM CFT	0.984	0.955–1.014	0.29
EXTEM MCF	1.002	0.995–1.009	0.64
EXTEM alpha	0.986	0.917–1.059	0.70

*aPTT* activated partial thromboplastin time, *CCT* conventional coagulation test, *CFT* clot formation time, *INR* international normalized ratio, *MCF* maximum clot firmness, *ROTEM* rotational thrombelastometry

#### Subgroup analysis of ACLF group with and without bleeding events and infectious diseases

In addition, differences in coagulation parameters were analyzed in patients with ACLF subgrouped by bleeding (*n* = 9) and without bleeding events (*n* = 13) (Table 7). The bleeding group showed significantly lower A10 in all tests (NATEM, INTEM and EXTEM), significantly lower MCF in NATEM and INTEM (Supplemental Table 1) and significantly lower fibrinogen (1.5 g/l vs. 1.9 g/l, *p* = 0.05) when compared with non-bleeding group. Platelets did not differ between the groups (Table 1). ACLF patients stratified by infectious diseases (*n* = 14) and without infectious diseases (*n* = 8) revealed significantly lower PT (Quick) (40.0% vs. 51.3%, *p* = 0.04) and significantly higher von-Willebrand antigen (516.7% vs. 284.7%, *p* = 0.001). No differences were seen in ROTEM analysis (Supplemental Table 2 + 3).

**Table 7:** Laboratory findings and blood coagulation tests of subjects with ACLF stratified by bleeding events. Median (IQR) is shown with *p*-values for the group differences based on Mann-Whitney U test

	Limits of normal	No bleeding *n* = 13	% outside the limitsof normal	Bleeding *n* = 9	% outside the limitsof normal	***p***-value*
**Laboratory findings**						
Hemoglobin	13–17 g/dl	9.2 (2.05)	92.3%	9.0 (3.2)	100%	0.55
Creatinine	0.6–1.2 mg/dl	2.39 (2.4)	84.6%	1.6 (2.5)	77.8%	0.47
Bilirubin	< 1.0 mg/dl	3.1 (20.6)	76.9%	4.1 (9.7)	100%	0.43
AST	< 46 U/l	62.0 (44.0)	61.5%	42.5 (9.7)	22.2%	0.36
ALT	< 50 U/l	27.0 (42.0)	18.2%	27.0 (65.5)	14.3%	0.44
GGT	< 60 U/l	92.0 (119.0)	69.2%	71.0 (144.0)	55.6%	0.29
Alkaline Phosphatase	40–130 U/l	106.0 (111.0)	36.4%	97.5 (70.3)	11.1%	1.0
Albumin	30–50 g/l	31.0 (7.55)	36.4%	30.2 (6.03)	57.1%	0.64
**Coagulation tests**						
INR	< 1.2	1.7 (0.9)	84.6%	1.4 (0.6)	77.8%	0.64
PT	70–120%	40.0 (30)	100%	0.51 (0.2)	44.4%	0.70
aPTT	< 36 s	32.9 (15.4)	30.8%	30.2 (27.6)	22.2%	0.85
Platelet count	150–440/nl	72.5 (75.3)	23.1%	57.0 (40.5)	44.4%	0.32
Fibrinogen	1.8–3.5 g/l	1.9 (1.6)	38.5%	1.5 (0.8)	77.8%	**0.05**
Antithrombin III-activity	80–120%	45.7 (41.0)	92.3%	39.7 (28.0)	100%	0.47
Thrombin time	< 22 s	20.2 (3.2)	15.4%	20.5 (8.9)	44.4%	0.16
Protein C	60–120%	32.3 (43.0)	76.9%	36.4 (27.0)	77.8%	0.74
Protein S (free)	>80%	51.8 (42.0)	76.9%	57.4 (27.0)	88.9%	1.0
Factor V	70–120%	46.0 (37.0)	92.3%	37.0 (26.0)	88.9%	0.51
Factor VIII	80–120%	255.5 (244.0)	100%	209.7 (116.0)	88.9%	0.96
von-Willebrand antigen	70–120%	456.9 (210.0)	100%	353.6 (337.0)	100%	0.23
ADAMTS13 activity	40–130%	81.9 (27.0)	0.0%	74.3 (57.0)	22.2%	0.34

*ACLF* acute-on-chronic liver failure, *ADAMTS13* desintegrin and metalloprotease with thrombospondin-1-like domains 13, *ALT* alanine aminotransferase, *AST* aspartate aminotransferase, *GGT* gamma GT, *INR* international normalized ratio, *aPTT* activated partial thromboplastin time, *PT* prothrombin time

## Discussion

Patients with liver cirrhosis typically show abnormal coagulation parameters in CCT. As it has been suggested that ROTEM gives a better overview of the coagulation system in liver cirrhosis, the aim of our study was to investigate hemostasis in Non-ACLF and ACLF patients, to evaluate the agreement of both methods and to assessed prospectively the test performance based on clinical outcomes in ACLF patients.

As expected, Non-ACLF and ACLF patients differed significantly in the severity of disease (MELD score, Child Pugh und class, grade of ascites and hepatic encephalopathy) and length of ICU and hospital stay. This was reflected in more pathological laboratory findings (bilirubin, creatinine and hemoglobin) in the ACLF group. Results for INR/PT and platelets were outside the normal range in Non-ACLF and ACLF patients. This grade of derangement in coagulation based on CCT was more pronounced in ACLF than in Non-ACLF patients. It is known that in patients with liver cirrhosis, thrombocytopenia is rebalanced by increased levels of factor VIII [12] and von-Willebrand factor [4] and decreased levels of ADAMTS13 [13]. In our study cohort, this was observed in Non-ACLF and ACLF patients, too. Of note, ACLF patients showed more severe thrombocytopenia paralleled by a more pronounced elevation of von-Willebrand factor and lower ADAMTS13 levels, while there was no significant difference in factor VIII elevation between the groups. Comparable differences between patients with stable cirrhosis and ACLF have previously been seen in a study by Fisher et al. [14] However, in contrast to our study, in their study a significant difference was also seen for factor VIII levels. As von-Willebrand factor is known as non-invasive predictor for portal hypertension, hepatic decompensation and a marker for procoagulant imbalance, this significant elevation in ACLF patients maybe represents more severe illness in this cohort [15, 16].

In our study, ROTEM analysis revealed functionally normal hemostasis in Non-ACLF patients. All measured ROTEM parameters (NATEM, EXTEM, INTEM and FIBTEM) were within the normal range except for a slightly prolonged CFT in INTEM. This observation can be interpreted as a ‘rebalanced’ coagulation state in Non-ACLF patients. Compared to the Non-ACLF group, the ACLF patients showed significantly more compromised values in nearly all measured ROTEM parameters (INTEM, EXTEM, FIBTEM and APTEM), in particular values for A10 and MCF, which represent maximum clot firmness. Abnormal MCF can be interpreted as the beginning of an imbalanced state in ACLF patients, especially with respect to clot stability. Hypo-functional clot stability tested via thrombin generation analysis was also reported previously for ACLF patients [14] and for acutely ill patients with severe chronic illness [17]. The strength of agreement for CCT and ROTEM analysis was determined to be fair for Non-ACLF patients and moderate for ACLF patients. Based on our results one could assume that particularly in ACLF patient’s transfusion policy guided by ROTEM results instead of using CCT could lead to much lower transfusion requirements.

Although a previous prospective trial in patients with liver cirrhosis and abnormal CCT showed that invasive procedures can be performed and non-variceal upper GI-bleeding can be treated with a lower requirement for blood products when guided by ROTEM results [18, 19], this approach is currently not used in standard clinical care settings. Overall survival was associated with PT, INR, and aPTT. In contrast to CCT parameters most ROTEM test parameters were not associated with overall survival. For ROTEM parameters, only EXTEM CFT and EXTEM alpha angle which represent extrinsic coagulation pathway were identified as important prognostic factors indicating that ROTEM is more suitable as point of care (POC) test to assess coagulation than as a prognostic marker for survival.

As bleeding events are a serious complication in cirrhotic patients and the bleeding risk increases in the state of decompensation, it appears to be important to have a prognostic marker in predicting bleeding events, especially in ACLF patients. In our study, bleeding events occurred significantly more often in the ACLF group. In this subgroup ROTEM parameters revealed an increased hemostatic imbalance, represented in significantly reduced A10 and MCF, paralleled by lower fibrinogen levels. When comparing patients with variceal bleeding and with non-variceal bleeding, no significant differences in all analysed ROTEM parameters could be seen (data not shown). This might be due to the small group size, but may hint to a general underlying etiology of a bleeding event. One might speculate that the event “bleeding” is occurring in ACLF patients when blood coagulation is deranged behind a critical threshold that might be reflected in A10 and MCF. Especially this aspect justify holisitic blood coagulation assays and make them a particularly interesting tool to evaluate, whether an ACLF patient is still in a rebalanced state or already in a decompensated state.

Patients with cancer often show a hypercoagulable state, and thrombotic events are common. There are only a few and inconsistent data on the hypercoagulability of patients with liver malignancies analyzed by ROTEM. Recent studies have revealed that HCC disturbs hemostatic balance and leads to a hypercoagulable state measured by a shorter CT (EXTEM, FIBTEM and INTEM) and a higher MCF (INTEM and FIBTEM) [20–22]. MCF FIBTEM > 25 mm was associated with a 5-fold increase in PVT risk. In our cohort hypercoagulability was not detected [21].

Limitation of the study is the small cohort size, especially in the subgroup analysis of bleeding events in the ACLF group. Therefore, further studies have to follow with a larger number of patients. We - like other groups before - demonstrated that ROTEM is superior for assessing hemostasis in Non-ACLF and ACLF patients. In addition to that, our research revealed, that the ROTEM parameters A10 and MCF represent suitable prognostic parameters to identify ACLF patients, who are at risk for bleeding events. Bleeding events are a severe complication in cirrhotic patients with increased mortality. If one could prevent this complication, the prognosis and long-term survival would be improved in this cohort.

In conclusion, our study highlights the advantages of ROTEM for assessing hemostasis in Non-ACLF and ACLF patients. CCT for both groups showed an imbalanced state, whereas ROTEM demonstrated a balanced hemostasis in Non-ACLF patients and a beginning imbalance in ACLF patients. The strength of agreement between the methods was poor for both groups. This discrepancy shows the need for further studies to emphasize the importance of ROTEM in clinical practice.

## Supplementary information

**Additional file 1 Supplemental Table 1**. ROTEM analysis of subjects with ACLF stratified by bleeding events. Median (IQR) is shown with *p*-values for the group differences based on Mann-Whitney U-test. **Supplemental Table 2**. Laboratory findings and blood coagulation tests of subjects with ACLF stratified by infectious disease. Median (IQR) is shown with *p*-values for the group differences based on Mann-Whitney U-test. **Supplemental Table 3**. ROTEM analysis of subjects with ACLF stratified by infectious disease. Median (IQR) is shown with *p*-values for the group differences based on Mann-Whitney U-test.

## Data Availability

The dataset used and analyzed during the current study is available from the corresponding author on reasonable request.

## Abbreviations

ACLF: Acute-on-chronic liver failure
aPTT: Activated partial thromboplastin time
PPSB: Prothrombin complex concentrates
CCT: Conventional coagulation test
CFT: Clot formation time
CT: Clotting time
ERCP: Endoscopic retrograde cholangiopancreaticography
FFP: Fresh frozen plasma
HCC: Hepatocellular carcinoma
ICU: Intensive care unit
INR: International normalized ratio
IQR: Interquartile range
MCF: Maximal clot firmness
MELD: Model of end stage liver disease
ML: Maximum lysis
ROTEM: Rotational thrombelastometry
SIRT: Selective internal radiation therapy
TACE: Transarterial chemoembolization
TEG: Thrombelastography
TIPSS: Transarterial portosystemic shunt
TACP: Transarterial chemoperfusion
