# Supplementary material for: Rotational thrombelastometry (ROTEM) improves hemostasis assessment compared to conventional coagulation test in ACLF and Non-ACLF patients
Source: BMC Gastroenterol. 2020 Aug 17;20:271. doi: 10.1186/s12876-020-01413-w (PMC7433140; doi:10.1186/s12876-020-01413-w)
Supplement: Supplementary file 1 — Additional file 1 Supplemental Table 1. ROTEM analysis of subjects with ACLF stratified by bleeding events. Median (IQR) is shown with p-values for the group differences based on Mann-Whitney U-test. Supplemental Table 2. Laboratory findings and blood coagulation tests of subjects with ACLF stratified by infectious disease. Median (IQR) is shown with p-values for the group differences based on Mann-Whitney U-test. Supplemental Table 3. ROTEM analysis of subjects with ACLF stratified by infectious disease. Median (IQR) is shown with p-values for the group differences based on Mann-Whitney U-test. [file 12876_2020_1413_MOESM1_ESM.docx]

Supplemental Table 1 ROTEM analysis of subjects with ACLF stratified by bleeding events. Median (IQR) is shown with p-values for the group differences based on Mann-Whitney U-test.

|  | **Limits of normal** | **No bleeding**  n=13 | **% outside the limits of normal** | **Bleeding**  n=9 | **% outside the limits of normal** | **p-value** |
| --- | --- | --- | --- | --- | --- | --- |
| **NATEM** |  |  |  |  |  |  |
| CT | 300-1000 s | 650.0 (398.0) | 15.4% | 650.5 (173.8) | 0.0% | 1.0 |
| CFT | 150-700 s | 194.0 (89.5) | 0.0% | 245.0 (184.0) | 0.0% | 0.14 |
| MCF | 40-65 mm | 49.0 (16.5) | 23.1% | 36.5 (13.5) | 62.5% | **0.045** |
| A10 | - | 39.0 (14.0) | - | 30.5 (11.0) | - | **0.01** |
| α-angle | 30-70° | 57.0 (9.0) | 0.0% | 52.0 (19.3) | 0.0% | 0.27 |
| **INTEM** |  |  |  |  |  |  |
| CT | 100-240 s | 235.0 (54.0) | 23.1% | 188.0 (156.0) | 12.5% | 0.37 |
| CFT | 30-110 s | 137.0 (110.0) | 53.8% | 40.0 (15.0) | 87.5% | **<0.0001** |
| MCF | 50-72 mm | 45.0 (20.0) | 30.8% | 65.0 (16.0) | 37.5% | **0.045** |
| A10 | 44-66 s | 38.0 (19.5) | 61.5% | 33.0 (13.0) | 87.5% | **<0.0001** |
| α-angle | 70-83° | 67.0 (12.0) | 53.8% | 9.0 (11.0) | 75.0% | 0.76 |
| **EXTEM** |  |  |  |  |  |  |
| CT | 38-79 s | 71.0 (18.0) | 15.4% | 76.0 (24.0) | 33.3% | 0.32 |
| CFT | 34-159 s | 150.0 (128.0) | 46.2% | 229.0 (116.0) | 77.8% | 0.05 |
| MCF | 50-72 mm | 47.0 (21.0) | 15.4% | 40.0 (12.0) | 44.4% | 0.08 |
| A10 | 43-65 s | 39.0 (24.0) | 61.5% | 32.0 (9.0) | 88.9% | **0.50** |
| α-angle | 63-83° | 66.0 (17.5) | 15.4% | 58.0 (16.5) | 55.6% | 0.14 |
| **FIBTEM** |  |  |  |  |  |  |
| MCF | 9-25 mm | 11.0 (12.0) | 30.8% | 9.0 (4.5) | 33.3% | 0.26 |
| A10 | - | 11.0 (11.0) | - | 8.0 (3.0) | - | 0.14 |
| **APTEM** |  |  |  |  |  |  |
| CT | 35-80 s | 64.0 (18.0) | 15.4% | 66.0 (25.5) | 22.2% | 0.29 |
| CFT | 35-160 s | 136.0 (127.5) | 46.2% | 202.0 (93.5) | 77.8% | 0.71 |
| MCF | 53-72 mm | 47.0 (19.5) | 15.4% | 42.0 (11.5) | 33.3% | 0.10 |
| A10 | - | 40.0 (21.5) | - | 34.0 (8.5) | - | 0.13 |
| α-angle | - | 64.0 (18.0) | - | 61.0 (15.5) | - | 0.16 |

A10. amplitudes at 10 minutes; ACLF. acute-on-chronic liver failure; CFT. clot formation time; CT. clotting time; IQR. interquartile range; MCF. maximum clot firmness; ML. maximal lysis; SD. standard deviation;

Supplemental Table 2 Laboratory findings and blood coagulation tests of subjects with ACLF stratified by infectious disease. Median (IQR) is shown with p-values for the group differences based on Mann-Whitney U-test.

|  | **Limits of normal** | **No Infection**  n=8 | **% outside the limits of normal** | **Infection**  n=14 | **% outside the limits of normal** | **p-value** |
| --- | --- | --- | --- | --- | --- | --- |
| **Laboratory findings** |  |  |  |  |  |  |
| Hemoglobin | 13-17 g/dl | 8.5 (5.1) | 87.5% | 8.9 (2.2) | 100% | 0.37 |
| Creatinine | 0.6-1.2 mg/dl | 2.2 (2.9) | 75% | 2.1 (1.4) | 14.3% | 0.87 |
| Bilirubin | <1.0 mg/dl | 3.9 (2.6) | 12.5% | 3.4 (24.3) | 14.3% | 0.37 |
| AST | <46 U/l | 40.0 (15.5) | 100% | 72.5 (54.3) | 28.6% | **0.005** |
| ALT | <50 U/l | 20.0 (13.5) | 75% | 34.5 (41.7) | 75% | 0.39 |
| GGT | <60 U/l | 64.0 (53.5) | 37.5% | 108.0 (141.3) | 35.7% | 0.68 |
| Alkaline Phosphatase | 40-130 U/l | 100.0 (78.0) | 83.3% | 125.5 (97.0) | 66.7% | 0.24 |
| Albumin | 30-50 g/l | 32.5 (9.7) | 40% | 31.0 (4.1) | 30.8% | 1.0 |
| **Coagulation tests** |  |  |  |  |  |  |
| INR | <1.2 | 1.4 (0.3) | 62.5% | 1.7 (0.9) | 92.9% | **0.04** |
| PT (Quick) | 70-120% | 51.3 (21.0) | 87.5% | 40.0 (25.0) | 100% | **0.04** |
| aPTT | <36 s | 30.2 (9.9) | 12.5% | 34.8 (19.3) | 42.9% | 0.19 |
| Platelet count | 150-440/nl | 52.0 (15.0) | 100% | 72.5 (83.0) | 85.7% | 0.19 |
| Fibrinogen | 1.8-3.5 g/l | 1.6 (0.9) | 50% | 1.6 (1.3) | 57.1% | 0.62 |
| Antithrombin III-activity | 80-120% | 43.3 (20.0) | 100% | 34.7 (38.0) | 92.9% | 0.48 |
| Thrombin time | <22 s | 19.5 (8.4) | 37.5% | 20.6 (3.4) | 14.3% | 0.87 |
| Protein C | 60-120% | 37.8 (36.0) | 75% | 27.4 (28.0) | 78.6% | 0.21 |
| Protein S (free) | >80% | 66.9 (34.0) | 62.5% | 50.4 (30.0) | 85.7% | 0.17 |
| Factor V | 70-120% | 52.0 (43.0) | 75% | 36.4 (28.0) | 78.6% | 0.24 |
| Factor VIII | 80-120% | 174.3 (60.0) | 87.5% | 270.5 (138.0) | 100% | 0.13 |
| von-Willebrand antigen | 70-120% | 284.7 (118.0) | 100% | 516.7 (217.0) | 100% | **0.001** |
| ADAMTS13 activity | 40-130% | 81.1 (27.0) | 0% | 80.3 (50.0) | 14.3% | 0.54 |

ACLF. acute-on-chronic liver failure; ADAMTS13. desintegrin and metalloprotease with thrombospondin-1-like domains 13; ALT. alanine aminotransferase. AST. aspartate aminotransferase. GGT. gamma GT; INR. international normalized ratio; IQR. interquartile range; aPTT. activated partial thromboplastin time; PT. prothrombin time;

Supplemental Table 3 ROTEM analysis of subjects with ACLF stratified by infectious disease. Median (IQR) is shown with p-values for the group differences based on Mann-Whitney U-test.

|  | **Limits of normal** | **No infection**  n=8 | **% outside the limits of normal** | **Infection**  n=14 | **% outside the limits of normal** | **p-value** |
| --- | --- | --- | --- | --- | --- | --- |
| **NATEM** |  |  |  |  |  |  |
| CT | 300-1000 s | 662.0 (268.0) | 14.3% | 615.0 (243.5) | 7.1% | 0.15 |
| CFT | 150-700 s | 237.0 (110.0) | 0% | 203.0 (130.0) | 0% | 0.29 |
| MCF | 40-65 mm | 37.0 (16.0) | 57.1% | 45.0 (16.0) | 28.6% | 0.40 |
| A10 | - | 31.0 (16.0) | - | 37.5 (13.5) | - | 0.40 |
| α-angle | 30-70° | 53.0 (16.0) | 0% | 55.0 (12.8) | 0% | 0.54 |
| **INTEM** |  |  |  |  |  |  |
| CT | 100-240 s | 203.0 (66.0) | 12.5% | 215.0 (58.3) | 14.3% | 0.86 |
| CFT | 30-110 s | 212.0 (44.0) | 85.7% | 129.0 (100.0) | 42.9% | 0.08 |
| MCF | 50-72 mm | 39.0 (2.0) | 71.4% | 47.0 (16.5) | 21.4% | 0.07 |
| A10 | 44-66 s | 33.0 (1.0) | 100% | 39.0 (18.0) | 71.4% | **0.046** |
| α-angle | 70-83° | 62.0 (15.0) | 71.4% | 67.5 (11.5) | 28.6% | 0.17 |
| **EXTEM** |  |  |  |  |  |  |
| CT | 38-79 s | 71.5 (30.0) | 25% | 72.0 (12.0) | 21.4% | 0.97 |
| CFT | 34-159 s | 220.5 (60.0) | 87.5% | 144.5 (119.5) | 35.7% | 0.19 |
| MCF | 50-72 mm | 40.0 (6.8) | 37.5% | 48.0 (20.5) | 21.4% | 0.45 |
| A10 | 43-65 s | 32.0 (6.3) | 87.5% | 39.0 (19.5) | 64.3% | 0.19 |
| α-angle | 63-83° | 59.0 (14.0) | 62.5% | 66.5 (19.0) | 57.1% | 0.44 |
| **FIBTEM** |  |  |  |  |  |  |
| MCF | 9-25 mm | 9.0 (5.8) | 50% | 11.0 (10.8) | 28.6% | 0.27 |
| A10 | - | 8.0 (5.5) | - | 9.0 (9.8) | - | 0.21 |
| **APTEM** |  |  |  |  |  |  |
| CT | 35-80 s | 78.0 (32.5) | 37.5% | 63.0 (9.8) | 7.1% | 0.24 |
| CFT | 35-160 s | 202.5 (41.5) | 87.5% | 136.0 (124.3) | 42.9% | 0.19 |
| MCF | 53-72 mm | 41.5 (6.3) | 12.5% | 48.5 (18.3) | 21.4% | 0.10 |
| A10 | - | 34.0 (4.3) | - | 40.5 (19.3) | - | 0.21 |
| α-angle | - | 61.0 (13.3) | - | 65.0 (19.3) | - | 0.30 |

A10. amplitudes at 10 minutes; ACLF. acute-on-chronic liver failure; CFT. clot formation time; CT. clotting time; IQR. interquartile range; MCF. maximum clot firmness; ML. maximal lysis;
